# The diagnosis and management of ectopic thyroid cancer: a systematic review

**DOI:** 10.3389/fendo.2026.1856987

**Published:** 2026-05-29

**Authors:** Min Quan, Xuerui Tao, Jiayuan Tang, Hong Chen, Qiuhan Liu, Qiaohua Li, Jiayao Li, Xiyi Wang, Wenjun Liao, Chao Li, Shichuan Zhang, Yue Zhao

**Affiliations:** 1 Department of Radiation Oncology, Radiation Oncology Key Laboratory of Sichuan Province, Sichuan Clinical Research Center for Cancer, Sichuan Cancer Hospital & Institute, Chengdu, China; 2 School of Medical and Life Sciences, Chengdu University of Traditional Chinese Medicine, Chengdu, China; 3 School of Medicine, University of Electronic Science and Technology of China, Chengdu, China; 4 Department of Integrated Chinese and Western Medicine, Sichuan Clinical Research Center for Cancer, Sichuan Cancer Hospital & Institute, Chengdu, China; 5 School of Basic Medical Sciences, Chengdu University of Traditional Chinese Medicine, Chengdu, China; 6 Department of Head and Neck Surgery, Sichuan Clinical Research Center for Cancer, Sichuan Cancer Hospital & Institute, Chengdu, China

**Keywords:** ectopic thyroid cancer, papillary thyroid carcinoma, systematic review, thyroglossal duct cyst, thyroidectomy

## Abstract

**Background:**

Ectopic thyroid carcinoma (ETC) is a rare malignancy for which standardized treatment guidelines remain unavailable. This systematic review aimed to comprehensively characterize the clinical features, treatment patterns, outcomes, and prognostic factors of ETC using the largest case-based dataset reported to date.

**Methods:**

A systematic literature search of PubMed, Scopus, and Web of Science was conducted from database inception to May 27, 2025. A total of 348 eligible reports were included. Data from 506 patients with ETC were extracted and analyzed, including demographics, tumor pathology, treatment strategies, recurrence, and survival outcomes.

**Results:**

The study included 506 patients, with a female-to-male ratio of 1.7:1 and a mean age of 41.6 years. Papillary thyroid carcinoma was the predominant histological subtype, accounting for 85.6% of cases. Most patients presented with localized disease (77.9%). Surgery was the primary treatment method (98.8%), and 40.6% of patients also received radioiodine therapy. Multivariable Cox regression showed that age >50 years (HR = 3.75, 95% CI: 1.92–7.32, P < 0.001) and ectopic tumor location in the chest, abdomen, or pelvis (HR = 7.23, 95% CI: 2.61–20.04, P < 0.001) were independently associated with worse overall survival. In the propensity-score-matched cohort, recurrence occurred in 3 of 50 patients who underwent orthotopic thyroidectomy and in 2 of 50 patients who did not, corresponding to recurrence rates of 6.0% and 4.0%, respectively. This difference was not statistically significant in paired analysis (McNemar P = 1.000).

**Conclusions:**

This systematic review provides the largest synthesis of ETC cases to date and identifies advanced age and non-cervical ectopic tumor location as adverse prognostic factors. Orthotopic thyroidectomy was not associated with reduced recurrence after propensity-score matching. However, given the small number of recurrence events and the case-based nature of the available evidence, this finding should be interpreted as hypothesis-generating rather than definitive. These results support a risk-adapted management strategy in which thyroidectomy is individualized rather than performed routinely.

**Systematic Review Registration:**

https://www.crd.york.ac.uk/prospero/, identifier CRD420250630606.

## Introduction

ETC is a rare malignancy arising from thyroid tissue that fails to migrate normally during embryonic development (1). Ectopic thyroid tissue may occur anywhere along the developmental migration pathway from the foramen cecum of the tongue to the lower neck and mediastinum, with the tongue, thyroglossal duct, and anterior neck being the most commonly reported sites (2, 3). Rare cases have also been described in unusual locations, including the heart (4), hepatic hilum (5), and ovary (6). ETC predominantly affects young women and appears to be more frequently reported in Asian populations (7, 8).

Similar to orthotopic thyroid tissue, ectopic thyroid tissue is susceptible to a range of pathological processes, including hyperplasia, inflammation, and malignant transformation (9). Malignant transformation is exceedingly rare, with an estimated incidence of approximately 1% (10). Histologically, papillary thyroid carcinoma (PTC), which arises from follicular epithelial cells, is the most common subtype. Other histological types, including follicular carcinoma, anaplastic carcinoma, and medullary carcinoma arising from parafollicular C cells, have also been reported. In the present review, 44 cases provided detailed PTC subtype information, including 9 classical variants, 25 follicular variants, 3 diffuse sclerosing subtype (DSS), and 7 tall cell subtype (TCS).

A major diagnostic challenge is distinguishing true ETC from metastatic thyroid carcinoma arising from an occult primary lesion in the orthotopic thyroid (11). Therefore, comprehensive evaluation of the orthotopic thyroid is essential before establishing a diagnosis of ETC. However, standardized guidelines for the diagnosis and management of ETC remain unavailable, resulting in substantial variability in clinical practice.

Given the rarity of ETC and the lack of consensus regarding its optimal management, large-scale evidence synthesis is needed to inform clinical decision-making. Here, we present the largest systematic review of ETC to date, comprising 506 cases. We summarize the clinical characteristics, anatomical distribution, treatment strategies, prognostic factors, and patterns of treatment failure associated with ETC. This review aims to provide a more robust evidence base for risk-adapted management and future therapeutic decision-making. The protocol was prospectively registered in PROSPERO and is publicly available (CRD420250630606).

## Materials and methods

### Literature search

This research was conducted in accordance with the 2020 PRISMA guidelines and the published frameworks and strategies for conducting systematic reviews of case reports to ensure methodological rigor (12–16). The protocol for this review was also registered prospectively with PROSPERO. We conducted the search on PubMed, Scopus, and Web of Science for English-limited publications from the inception of these databases through May 27, 2025, with the following keywords and MeSH terms: ((“Thyroid Neoplasm” OR “Thyroid Carcinoma” OR “Thyroid Cancer”) AND (“Ectopic”)), as well as ((“Thyroglossal Duct” OR “Thyroglossal Cyst”) AND (“Carcinoma” OR “Cancer” OR “Malignancy” OR “Neoplasm” OR “Tumor”)). The bibliographies of relevant articles were searched to identify other appropriate articles. Two reviewers independently evaluated titles and abstracts after duplicate removal. Full text manuscripts of potential candidates were assessed for final eligibility, with discrepancies resolved through consensus.

### Selection and eligibility criteria

A total of 506 patients with confirmed ETC were identified through the literature screening process. In this review, some patients with ETC had concomitant lesions in the orthotopic thyroid gland. Nevertheless, the ectopic lesions included in our analysis were confirmed to contain thyroid follicular tissue and lacked lymphoid tissue or preserved nodal architecture, supporting their interpretation as primary tumors arising from ectopic thyroid tissue rather than metastatic deposits. For the prognosis analysis, the following inclusion and exclusion criteria were applied.

#### Inclusion criteria

1. Pathologically confirmed diagnosis of ETC.
2. Availability of complete intervention and follow-up data with clearly documented clinical outcomes.
3. Reported recurrence or death as secondary to ETC.

#### Exclusion criteria

1. Lack of clear intervention or follow-up information.
2. Death or recurrence due to causes unrelated to ETC.

### Data extraction

Data were systematically extracted from the included reports using a standardized form. The extracted variables included: gender, age, tumor location, pathological type, presence of orthotopic thyroid gland, intervention, and follow-up outcomes. Data points explicitly reported in the original articles were recorded. Missing data were noted as such and excluded from the respective analyses.

### Statistical analysis

Statistical analyses were performed using SPSS version 26.0. Survival analyses were conducted for 335 patients with ETC with sufficient outcome data, including 321 patients with ETC located in the head and neck region and 269 patients with thyroglossal duct carcinoma (TGDC). Continuous and categorical variables were summarized using appropriate descriptive statistics.

Univariate Cox proportional hazards regression was first performed to evaluate the association between each variable and survival outcomes. Variables with a P value <0.05 in univariate analysis were subsequently entered into multivariable Cox proportional hazards models to identify independent prognostic factors in the overall ETC cohort and in the head and neck subgroup. Kaplan–Meier survival curves were generated using GraphPad Prism, and survival differences between groups were compared using the log-rank test. Data that were not explicitly reported in the original articles were excluded from the corresponding analyses.

To further address potential confounding by indication, a propensity-score-matched sensitivity analysis was performed using R version 4.3.0. Propensity scores were estimated using logistic regression incorporating age, sex, tumor size, initial disease status, and pathological type. Because residual imbalance in age and sex was observed after conventional nearest-neighbor matching, exact matching by age group and sex was additionally applied. One-to-one nearest-neighbor matching without replacement was then performed using a caliper width of 0.2 standard deviations of the propensity score. Covariate balance was assessed using standardized mean differences, with a standardized mean difference (SMD) <0.1 considered indicative of adequate balance. Tumor position was considered as a candidate covariate but was not included in the final propensity-score model because no variation remained after complete-case filtering.

### Data management and quality assurance

Data from the 348 included reports were independently extracted by two reviewers. Extracted information included demographic characteristics, clinicopathological features, treatment details, recurrence, and survival outcomes. The methodological quality of the included case reports and case series was assessed using the JBI critical appraisal tools, and the results are provided in the **Supplementary Data**. No report was excluded solely on the basis of a low JBI score and the quality assessment was used to contextualize the strength and limitations of the available evidence.

Associations between clinical variables, including age, sex, tumor location, initial disease status, surgical approach, adjuvant therapy, and recurrence, were analyzed in the overall cohort, the head and neck subgroup, and the TGDC subgroup. All extracted data were cross-checked, and discrepancies were resolved through discussion or consultation with a third reviewer.

## Results

**Figure 1:**
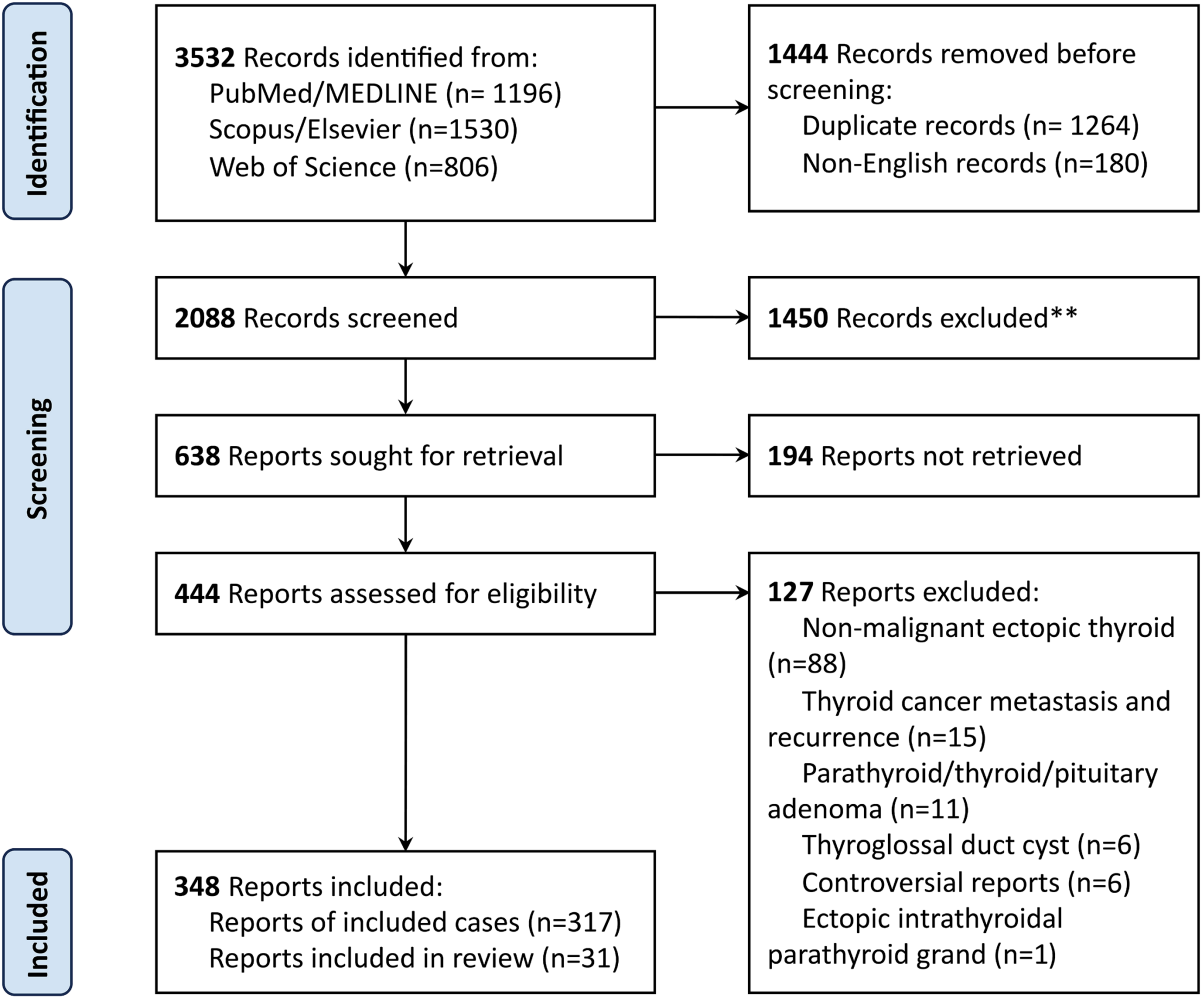
Literature flow diagram. **: study related to the thyroid cancer, parathyroid gland, thyroid cancer, thymoma, animal studies, non-thyroid tumors, surgical treatment of thyroglossal duct cysts, imaging diagnosis and evaluation of head and neck masses, pediatric head and neck lymphadenopathy and non-cases.

**Figure 2:**
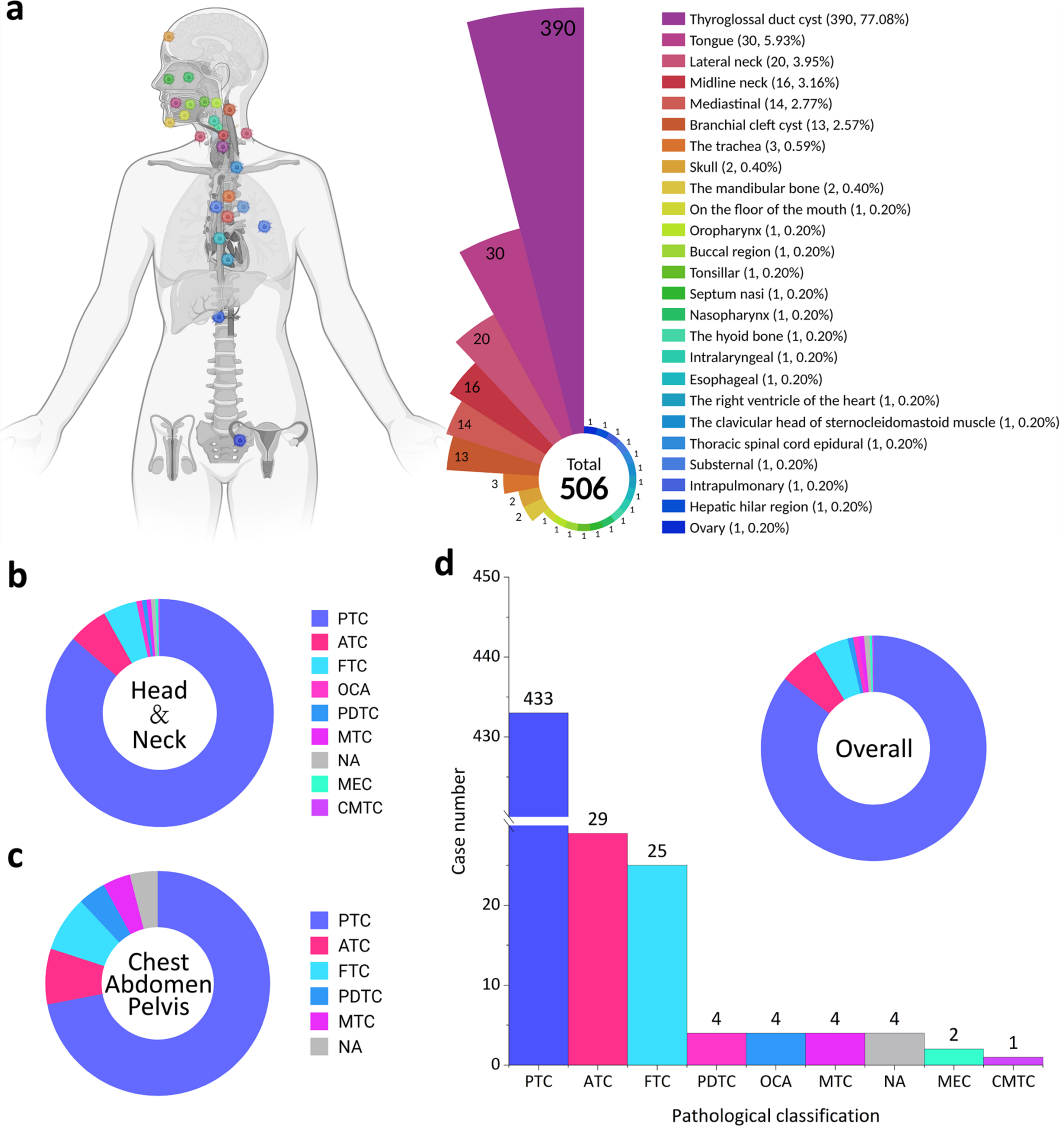
Anatomical and histopathological distribution of ectopic thyroid carcinoma. **(a)** Anatomical distribution of ETC according to tumor location. **(b)** Histopathological distribution in the head and neck ETC subgroup. **(c)** Histopathological distribution in the thoracic, abdominal, and pelvic ETC subgroup. **(d)** Histopathological distribution in the overall ETC cohort, shown as pie and bar charts. PTC, papillary thyroid carcinoma; FTC, follicular thyroid carcinoma; ATC, anaplastic thyroid carcinoma; PDTC, poorly differentiated thyroid carcinoma; OCA, oncocytic carcinoma; MTC, medullary thyroid carcinoma; MEC, mucoepidermoid carcinoma; CMTC, cribriform morular thyroid carcinoma; NA, not applicable.

The systematic literature search identified 3,532 records related to ETC and TGDC. After removal of 1,264 duplicates and exclusion of 180 non-English articles, 2,088 records underwent title and abstract screening. Of these, 1,450 were excluded as irrelevant, leaving 638 articles for full-text review. An additional 31 records were identified through manual screening of the reference lists of included reports. Ultimately, 348 articles met the eligibility criteria, comprising 506 patients with ETC (**Figure 1**). A comprehensive summary of tumor location, clinical manifestations, laboratory findings, imaging features, and pathological characteristics is provided (**Figures 2b-d**).

### Patient characteristics

This systematic review included 506 patients with ETC, of whom 315 were female (62.4%), 190 were male (37.6%) and sex was not reported in 1 case, corresponding to a female-to-male ratio of 1.7:1. The mean age was 41.6 ± 17.5 years, with a range of 8–97 years. Age data were available for 504 patients. Among them, 367 patients (72.8%) were aged ≤50 years and 137 patients (27.2%) were aged >50 years.

Orthotopic thyroid tissue was present in 447 patients (88.3%) and absent in 30 patients (5.9%). Histopathologically, papillary thyroid carcinoma was the most common subtype, accounting for 433 cases (85.6%), followed by anaplastic thyroid carcinoma in 29 cases (5.7%). At diagnosis, 394 patients (77.9%) had localized disease, whereas 112 patients (22.1%) presented with lymph node involvement or distant metastasis (**Table 1**).

**Table 1:** Baseline characteristics of patients with ectopic thyroid cancer.

N. patients included in our study	Number of patients	% of subgroup
Gender		
Female	315	62.3
Male	190	37.5
NA	1	0.2
Age (year)		
≤50	367	72.5
>50	137	27.1
NA	2	0.4
Orthotopic thyroid		
Yes	447	88.3
No	30	5.9
NA	29	5.7
Properties of the orthothyroid gland		
Benign	209	46.8
Malignant	102	22.8
NA	136	30.4
Thyroid function		
Normal	222	43.9
Hypothyroidism	14	2.8
Hyperthyroidism	6	1.2
NA	264	52.2
Pathological types of ectopic thyroid carcinoma		
PTC	433	85.6
ATC	29	5.7
FTC	25	4.9
PDTC	4	0.8
OCA	4	0.8
MTC	4	0.8
MEC	2	0.4
CMTC	1	0.2
NA	4	0.8
Ectopic sites		
Head and neck	481	95.1
Chest	23	4.5
Abdomen and pelvis	2	0.4
Disease stage at diagnosis		
Local	394	77.9
Metastatic	112	22.1
Clinical course		
No recurrence	330	65.2
Recurrence or died	46	9.1
NA	130	25.7

N, number of studies; NA, not applicable; PTC, papillary thyroid carcinoma; ATC, anaplastic thyroid carcinoma; FTC, follicular thyroid carcinoma; PDTC, poorly differentiated thyroid carcinoma; OCA, oncocytic carcinoma; MTC, medullary thyroid carcinoma; MEC, mucoepidermoid carcinoma; CMTC, cribriform morular thyroid carcinoma.

### Location distribution

The head and neck region was the predominant site of ETC, accounting for 481 cases (95.1%), followed by the chest in 23 cases (4.5%) and the abdomen/pelvis in 2 cases (0.4%). Within the head and neck region, the thyroglossal duct was the most common site, representing 390 cases (81.1%), followed by the tongue in 30 cases (6.2%) (**Figure 2a**). An orthotopic thyroid gland was present in 93.6% of evaluable head and neck cases.

Among thoracic ETC cases, the mediastinum was the most frequent location, accounting for 14 cases (60.9%). Other reported thoracic sites included the intratracheal lumen, esophagus, heart, and other rare locations, with most cases showing differentiated thyroid carcinoma histology (4, 8, 17–25).

### Clinical manifestations

Clinical presentation varied according to tumor location and size. In the head and neck region, the most common presentation was a neck or oral mass, observed in 346 of 481 patients (71.9%), and was typically painless. Associated symptoms included dysphagia (4.0%), voice changes (1.7%), and localized pain (1.2%). Among thoracic cases, 26.1% of patients were asymptomatic. Symptomatic thoracic cases presented with dysphagia, dyspnea, cough, or chest/back pain. Both abdominal/pelvic cases presented with pain.

### Laboratory tests

Thyroid function was documented in 242 patients, of whom 91.7% were euthyroid, 5.8% were hypothyroid, and 2.5% were hyperthyroid. Thyroglobulin (Tg) levels were available for 102 patients. Post-treatment Tg levels were within the normal range in 72 patients (70.6%), whereas elevated post-treatment Tg was observed in 15 patients (14.7%). Among patients with elevated post-treatment Tg, 6 of 15 (40.0%) experienced recurrence.

### Image evaluation

Although ectopic thyroid tissue is rare, imaging evaluation is recommended in suspected cases to distinguish ETC from metastatic lymph nodes and other malignancies. Commonly used imaging methods include ultrasound, computed tomography (CT), magnetic resonance imaging (MRI), and radionuclide imaging, often supplemented by thyroid function testing (8, 26–29).

Ultrasound was the most frequently used diagnostic method (50.0%), followed by CT (37.0%), radionuclide imaging (12.6%), and MRI (8.1%). Endoscopy was commonly used for tumors involving the airway or upper digestive tract, whereas PET/CT was generally reserved for complex cases. Evaluation of the orthotopic thyroid was performed primarily using ultrasound (51.9%), followed by radionuclide scanning (28.4%) and CT (22.4%).

### Pathology

When imaging findings were inconclusive, fine-needle aspiration cytology (FNAC) was useful for guiding management and avoiding unnecessary postoperative complications (30, 31). According to the 5th edition of the WHO Classification of Tumors of Endocrine Organs, papillary thyroid carcinoma was the most frequent pathological subtype, followed by anaplastic thyroid carcinoma and follicular thyroid carcinoma (**Figures 2b–d**) (32–34).

Immunohistochemical data were available for 69 patients. Most tumors showed positivity for TG, thyroid transcription factor-1 (TTF-1), cytokeratin 19 (CK19), galectin-3, and Hector Battifora mesothelial-1 (HBME-1). In anaplastic thyroid carcinoma, immunopositivity for TG, TTF-1, and cytokeratin AE1/AE3 was also reported. Genetic profiling was available for 16 patients, among whom *BRAF V600E* mutations were identified in 14 cases.

### Treatment strategies

**Figure 3:**
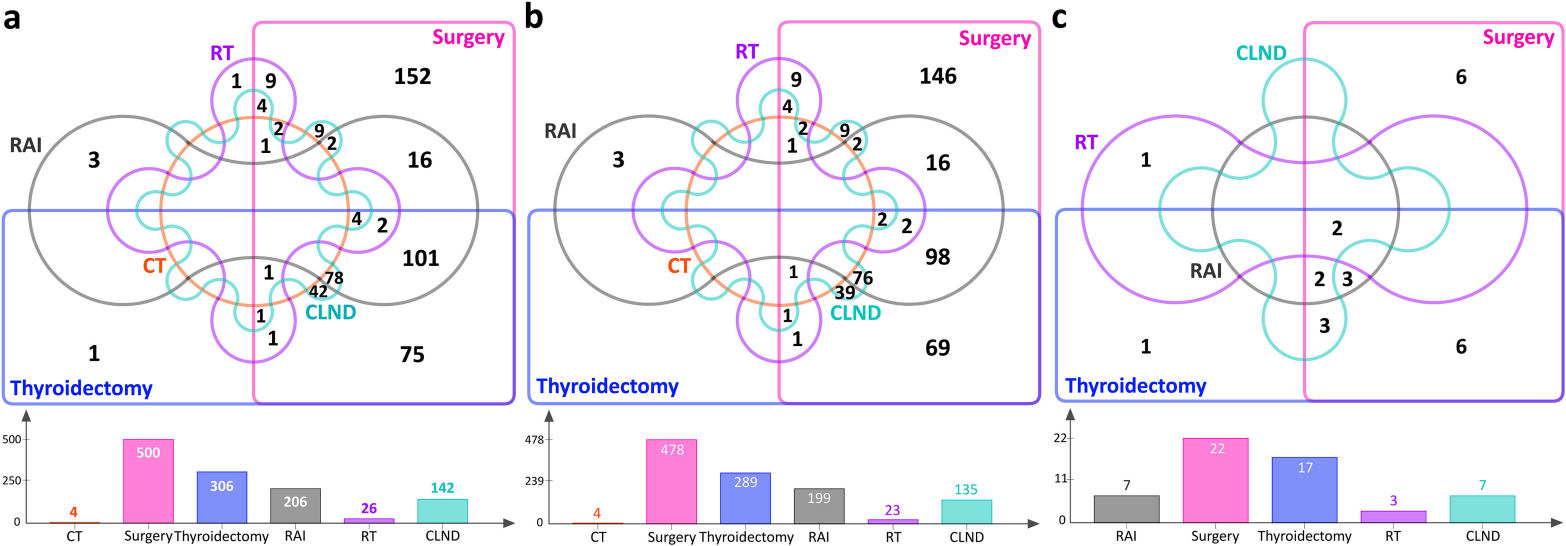
Edwards–Venn diagrams showing treatment combinations in patients with ectopic thyroid carcinoma. Edwards–Venn diagrams illustrate the distribution and overlap of treatment modalities in **(a)** the overall cohort, **(b)** the head and neck ETC subgroup, and **(c)** the thoracic, abdominal, and pelvic ETC subgroup. CLND, cervical lymph node dissection; CT, chemotherapy; RAI, radioiodine; RT, radiotherapy.

No consensus currently exists regarding the optimal management of ETC, although surgical resection remains the mainstay of treatment. Among the 506 patients included in this review, 500 underwent surgery as the initial treatment. Treatment methods among patients with available management data are summarized in **Figure 3a**.

In the head and neck region, TGDC is commonly treated by simple excision or the Sistrunk procedure. The latter has been associated with an approximately 25% higher 10-year survival rate than simple excision (35). Preoperative thyroid scintigraphy and cyst aspiration cytology are useful for surgical planning and positive findings may support modified Sistrunk surgery, such as wider excision margins, or total thyroidectomy (36). Surgical approaches for lingual thyroid carcinoma have been widely reported and include midline tongue-splitting with trans-oral resection, as well as external approaches such as trans-hyoid incision, trans-mandibular access, and lateral pharyngotomy with or without mandibular split. Tumor location and size are key determinants of the most appropriate surgical approach (37, 38).

Among 478 surgically treated head and neck cases, 196 patients (41.0%) received radioactive iodine (RAI), 23 (4.8%) received external-beam radiotherapy, and 4 (0.8%) received chemotherapy. Among 289 patients who underwent both ectopic tumor resection and orthotopic thyroidectomy, 102 (35.3%) had synchronous procedures and 173 (59.9%) underwent staged procedures (**Figure 3b**).

For thoracic ETC, reported surgical approaches include thoracotomy, sternotomy, and VATS (8). Among 25 non-head and neck cases, 7 patients received RAI and 3 received external-beam radiotherapy. Of the 16 patients who underwent concurrent thyroidectomy, 5 had synchronous surgery and 11 underwent staged procedures. One abdominal case was treated with thyroidectomy and lenvatinib, whereas one pelvic case underwent ectopic thyroid tumor resection followed by thyroidectomy and RAI therapy (**Figure 3c**).

The prognosis of ETC arising in thoracic, abdominal, or pelvic locations was generally poorer than that of head and neck ETC. Among 25 non-head and neck cases with follow-up data, 10 patients (40.0%) remained disease-free, 5 (20.0%) experienced recurrence, including 1 patient who died of pulmonary metastasis after cervical recurrence, 3 (12.0%) died, and 7 (28.0%) were lost to follow-up.

### Prognosis analysis in the overall cohort

As shown in the study flowchart (**Figure 1**), 348 reports were initially included, comprising 506 patients with ETC. Of these, 171 patients were excluded from the prognostic analysis because of uncertain prognosis (n = 130), unknown cause of death (n = 2), death from unrelated causes (n = 5), or unclear follow-up duration (n = 34). The final prognostic cohort therefore included 335 patients. The median follow-up duration was 27.0 months (95% CI: 23.42–30.58). Overall, the prognosis of ETC was favorable. Among the 335 patients included in the prognostic analysis, 299 (89.3%) had no evidence of recurrence, 29 (8.7%) experienced recurrence, and 7 (2.1%) died; 6 of these deaths occurred after recurrence.

In univariate and multivariable Cox regression analyses, sex, tumor size, initial disease status, presence of an orthotopic thyroid gland, orthotopic thyroidectomy, timing of orthotopic and ectopic thyroid surgery, orthotopic thyroid pathology, cervical lymph node dissection, RAI therapy, treatment method, and pathological type were not significantly associated with overall survival (all P > 0.05; **Table 2**).

**Table 2:** Prognostic factors associated with overall survival in the entire ETC cohort.

Variables	Cohort	Univariate analysis	Multivariate analysis		
		HR (95% CI)	P value	HR (95% CI)	P value
Age	≤50 vs. >50	4.118 (2.129-7.966)	<0.0001	3.747(1.919-7.318)	<0.0001
Gender	Female vs. Male	0.884 (0.447-1.746)	0.722		
Tumor size	>2 vs. ≤2	0.969 (0.291-3.228)	0.959		
Position	Chest/abdomen/pelvis vs. Head/neck	9.483 (3.536-25.430)	0.000	7.230(2.609-20.038)	0.000
Initial state	Metastatic vs. Local	1.741 (0.890-3.408)	0.106		
Orthotopic thyroid gland	Present vs. Absent	1.560 (0.476-5.113)	0.462		
Orthothyroidectomy	Resected vs. Non-resected	1.358 (0.640-2.883)	0.426		
Orthotopic and ectopic thyroid surgery	Concurrent vs. Staged	0.612 (0.215-1.741)	0.357		
Orthotopic thyroid pathology	Malignant vs. Benign	1.100 (0.506-2.393)	0.810		
CLND	With neck dissection vs. Without	1.159 (0.578-2.326)	0.677		
RAI	RAI therapy vs. No RAI therapy	0.825 (0.428-1.590)	0.565		
Treatment mode	Simple vs. Comprehensive	0.732 (0.370-1.446)	0.369		
Pathological type	PTC vs. Others	1.550 (0.645-3.726)	0.328		

HR, hazard ratio; 95% CI: 95% confidence interval; CLNA: cervical lymph node dissection; RAI, radioactive iodine; PTC, papillary thyroid carcinoma.

**Figure 4:**
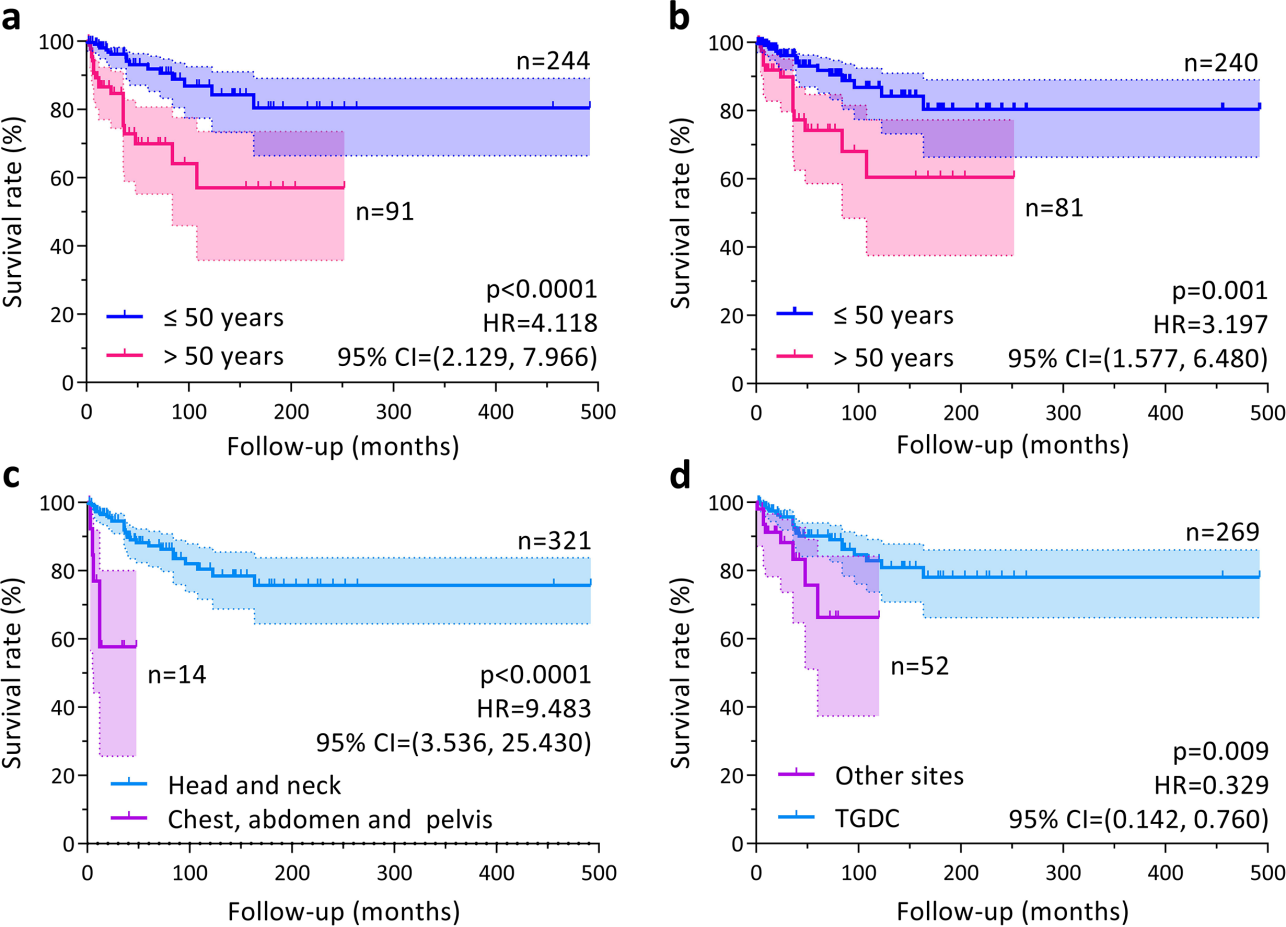
Kaplan–Meier survival analysis of ectopic thyroid carcinoma according to age and tumor location. Kaplan–Meier curves showing overall survival stratified by age in **(a)** the entire cohort and **(b)** the head and neck subgroup, and by tumor location in **(c)** the entire cohort and **(d)** the head and neck subgroup. HRs and 95% CIs were derived from univariate Cox proportional hazards regression analyses.

In univariate Cox regression analysis, age >50 years (HR = 4.12, 95% CI: 2.13–7.97, P < 0.001) and non-head and neck ectopic tumor location, defined as thoracic, abdominal, or pelvic location, were significantly associated with worse overall survival (HR = 9.48, 95% CI: 3.54–25.43, P < 0.001). The corresponding Kaplan–Meier curves are shown in **Figure 4**. And in multivariable Cox regression analysis, both variables remained independently associated with overall survival. Patients aged >50 years had a 3.75-fold higher risk of death than those aged ≤50 years (HR = 3.75, 95% CI: 1.92–7.32, P < 0.001). Similarly, patients with ETC located in the chest, abdomen, or pelvis had a 7.23-fold higher risk of death than those with head and neck ETC (HR = 7.23, 95% CI: 2.61–20.04, P < 0.001).

These findings suggest that prognosis in ETC is strongly influenced and heterogeneous by anatomical location. Head and neck ETC generally showed favorable outcomes, whereas thoracic, abdominal, and pelvic ETC were associated with substantially poorer survival. This may partly explain why initial disease status was not statistically significant in the overall cohort, as the metastatic category included both regional lymph node involvement and distant metastasis. Given the generally indolent behavior of PTC, regional lymph node metastasis alone may have limited impact on long-term overall survival.

### Prognostic analysis in head and neck ETC

Given the rarity of thoracic, abdominal, and pelvic ETC, we further analyzed prognostic factors in the head and neck subgroup to support risk-stratified treatment. A total of 321 patients with head and neck ETC met the inclusion criteria. Among them, 290 patients (90.3%) had no recurrence after treatment, whereas 31 patients (9.7%) experienced recurrence or metastasis.

In the prognostic analysis of 321 patients with head and neck ETC, age and tumor location were identified as independent prognostic factors. No significant associations with overall survival were observed for other variables, including sex, tumor size, initial metastatic status, or treatment methods. In univariate Cox regression analysis, age >50 years was significantly associated with worse overall survival (HR = 3.20, 95% CI: 1.58–6.48, P = 0.001). Tumor location was also significantly associated with prognosis. Patients with ETC arising from the thyroglossal duct had a lower risk of death than those with ETC at other head and neck sites (HR = 0.33, 95% CI: 0.14–0.76, P = 0.009). The corresponding Kaplan–Meier curves are shown in **Figure 4**.

Multivariable Cox regression analysis confirmed that both variables remained independently associated with overall survival. Patients aged >50 years had a 3.19-fold higher risk of death than those aged ≤50 years (HR = 3.19, 95% CI: 1.57–6.47, P = 0.001). In contrast, thyroglossal duct ETC was associated with a 67% lower risk of death compared with ETC at other head and neck sites, including the tongue, lateral neck, and tonsil (HR = 0.33, 95% CI: 0.14–0.77, P = 0.010, **Table 3**).

**Table 3:** Prognostic factors associated with overall survival in patients with head and neck ETC.

Variables	Group	Univariate analysis		Multivariate analysis	
		HR (95% CI)	P value	HR (95% CI)	P value
Age	≤50 vs. >50	3.197(1.577-6.480)	0.001	3.189(1.571-6.473)	0.001
Gender	Female vs. Male	1.183(0.566-2.470)	0.655		
Tumor size	>2 vs. ≤2	0.969(0.291-3.228)	0.959		
Position	Other sites vs. Thyroglossal duct	0.329(0.142-0.760)	0.009	0.330(0.142-0.766)	0.010
Initial state	Metastatic vs. Local	1.501(0.718-3.139)	0.281		
Orthotopic thyroid gland	Present vs. Absent	1.870(0.565-6.192)	0.306		
Orthothyroidectomy	Resected vs. Non-resected	1.330(0.579-3.054)	0.501		
Orthotopic and ectopic thyroid surgery	Concurrent vs. Staged	0.532(0.167-1.701)	0.288		
Orthotopic thyroid pathology	Malignant vs. Benign	0.844(0.369-1.931)	0.687		
Cervical lymph node dissection	With neck dissection vs. Without	1.083(0.517-2.269)	0.833		
RAI	RAI therapy vs. No RAI therapy	0.610(0.300-1.241)	0.172		
Treatment mode	Simple vs. Comprehensive	0.858(0.404-1.825)	0.691		
Pathological type	PTC vs. Others	1.159(0.405-3.314)	0.783		

HR, hazard ratio; 95% CI: 95% confidence interval; RAI, radioactive iodine; PTC, papillary thyroid carcinoma.

### Propensity-score-matched analysis of orthotopic thyroidectomy

In univariate Cox regression analysis, orthotopic thyroidectomy was not significantly associated with patient prognosis. To further evaluate this clinically relevant variable, propensity score matching (PSM) was performed using R version 4.3.0 to reduce potential confounding. Propensity scores were estimated using age, sex, tumor size, initial disease status, and pathological type, with exact matching additionally imposed on age group and sex. Covariate balance before and after matching is summarized in Appendix, **Supplementary Table 1**, and standardized mean differences are visualized using a Love plot in Appendix, **Supplementary Figure 1**.

In the unmatched cohort, recurrence occurred in 22 of 199 patients who underwent orthotopic thyroidectomy and in 10 of 94 patients who did not, corresponding to recurrence rates of 11.1% and 10.6%, respectively. After propensity-score matching with exact matching on age group and sex, 50 matched pairs were generated. All included covariates achieved adequate balance, with post-matching standardized mean differences below 0.1.

In the matched cohort, recurrence occurred in 3 of 50 patients in the orthotopic thyroidectomy group and in 2 of 50 patients in the non-thyroidectomy group, corresponding to recurrence rates of 6.0% and 4.0%, respectively. This difference was not statistically significant in paired analysis (McNemar P = 1.000). Recurrence outcomes before and after propensity score matching are presented in Appendix, **Supplementary Table 2**. Overall, orthotopic thyroidectomy was not associated with improved recurrence outcomes after adjustment for measured confounders. However, given the small number of events and the observational case-based nature of the evidence, this finding should be interpreted cautiously and regarded as hypothesis-generating.

### Summary of treatment failure cases

Follow-up data were available for 376 patients, among whom 40 experienced recurrence, including 9 patients who subsequently died. One patient without recurrence died of non-disease-related myocardial infarction, and 5 additional patients died during follow-up. Recurrences occurred predominantly in the head and neck region (87.5%, n = 35), including the thyroglossal duct (n = 25), tongue (n = 4), lateral neck (n = 2), mid-neck (n = 2), skull (n = 1), and oropharynx (n = 1). Thoracic recurrences accounted for 12.5% of cases (n = 5), involving the mediastinum (n = 2), thoracic spinal epidural space (n = 1), trachea (n = 1), and substernal region (n = 1). Deaths occurred in patients with tumors located in the mandible (n = 1), mediastinum (n = 2), porta hepatis (n = 1), and thyroglossal duct (n = 2) (Appendix, **Supplementary Table 3**, **4**).

The mean age at recurrence was >50 years, except among patients with TGDC, whose mean age at recurrence was <50 years. Papillary thyroid carcinoma was the predominant histological type among recurrence cases (n = 32), including five cases with high-risk or intermediate-to-high-risk variants characterized by aggressive biological behavior, namely the tall cell variant and diffuse sclerosing variant. Other histological types included anaplastic thyroid carcinoma (n = 4), follicular thyroid carcinoma (n = 2), oncocytic carcinoma (n = 1), and poorly differentiated thyroid carcinoma (n = 1). Among 31 patients with orthotopic thyroid tissue and available pathological data, 12 (38.7%) had malignant orthotopic thyroid lesions and 19 (61.3%) had benign lesions.

At diagnosis, 25 patients with recurrence (62.5%) had localized disease, whereas 15 (37.5%) had lymph node involvement or distant metastasis. *BRAF V600E* mutations were detected in 2 cases, and concurrent *BRAF V600E* and *TERT promoter C228T* mutations were detected in 1 case. The mean time to recurrence was 37.7 months, ranging from 1.0 to 163.4 months. Cervical lymph nodes were the most common recurrence site (50.0%, n = 20), followed by local recurrence (n = 10) and distant metastasis (n = 6). Among 14 patients who underwent lymph node dissection, recurrence occurred in 2 of 4 patients with pathologically negative nodes and in 3 of 10 patients with pathologically positive nodes.

These findings suggest that, in addition to age and tumor location, several factors may contribute to treatment failure and warrant further investigation, including the role of routine cervical lymph node dissection, the extent of lymph node resection, and the presence of aggressive histological subtypes.

Among the 6 disease-related deaths, the mean age was 76 years. Four patients had tumors in rare anatomical sites, including the mediastinum, mandible, and porta hepatis. Histological types included follicular thyroid carcinoma (n = 2), papillary thyroid carcinoma (n = 2), and anaplastic thyroid carcinoma (n = 2). Two patients presented with metastatic disease at diagnosis. Treatment approaches varied and were often limited. For example, one patient with mediastinal ETC underwent excisional biopsy only, whereas another received no treatment. Potential factors associated with mortality included advanced age, aggressive histology, rare tumor location, metastatic disease at diagnosis, and limited treatment.

## Discussion and limitations

To our knowledge, this study represents the largest systematic review and analysis of ETC to date, comprising 506 reported cases. This expanded case-based cohort enables a more comprehensive characterization of the epidemiology, clinical behavior, treatment patterns, and prognostic factors of this rare malignancy. Our findings confirm that ETC shares several pathological features with orthotopic thyroid carcinoma, particularly the predominance of papillary thyroid carcinoma, but also demonstrates distinct clinical behavior that is strongly influenced by patient age and tumor location. Importantly, orthotopic thyroidectomy was not associated with improved recurrence outcomes after propensity-score matching, suggesting that routine thyroidectomy should not be performed solely for presumed prognostic benefit.

Ectopic thyroid tissue is a rare developmental anomaly, most commonly attributed to aberrant thyroid migration during embryogenesis. Other entities, including thyroid tissue implantation after surgery and metastatic thyroid carcinoma, may mimic ectopic thyroid lesions and should be carefully distinguished (39). In the present review, most cases were considered to arise from ectopic thyroid tissue related to abnormal embryological migration followed by malignant transformation. However, differentiating primary ETC from metastatic thyroid carcinoma originating from an occult orthotopic primary remains a major diagnostic challenge. In addition to clinical presentation, imaging findings, and histopathological assessment, the presence of normal thyroid follicles and the absence of lymphoid tissue or nodal architecture within the ectopic lesion may support the diagnosis of primary ETC rather than nodal metastasis.

The clinical and pathological landscape observed in this review is consistent with the embryological distribution of ectopic thyroid tissue (40). A particularly important clinical consideration is the coexistence of ectopic and orthotopic thyroid tissue. Previous reports have suggested that many patients with ectopic thyroid have no normally located thyroid gland (31, 41), whereas in our cohort, an orthotopic thyroid gland coexisted with ETC in 88.3% of cases. Among patients with available orthotopic thyroid pathology, 102 had malignant lesions, underscoring the importance of comprehensive evaluation of both the ectopic lesion and the orthotopic thyroid gland. Accordingly, diagnostic assessment should be tailored to tumor location and should routinely include evaluation of the orthotopic thyroid to exclude synchronous malignancy or an occult primary tumor.

Surgical resection remains the cornerstone of ETC treatment, with 98.8% of patients in this review undergoing surgery (42). Prognostic analysis identified two key factors independently associated with worse overall survival. First, age >50 years was a strong adverse prognostic factor. Patients older than 50 years had a 3.75-fold higher risk of death than those aged ≤50 years (HR = 3.75, 95% CI: 1.92–7.32, P < 0.001). Second, non-head and neck ETC, including tumors located in the chest, abdomen, or pelvis, was independently associated with poorer prognosis. Patients with ETC in these regions had a 7.23-fold higher risk of death than those with head and neck ETC (HR = 7.23, 95% CI: 2.61–20.04, P < 0.001).

The tall cell variant accounts for approximately 9% of all PTC cases and is associated with higher rates of recurrence and disease-specific mortality. The diffuse sclerosing variant, which is more commonly observed in younger patients, also exhibits aggressive clinical behavior and is associated with a higher risk of distant metastasis (21, 43). In contrast, the follicular variant generally shows less aggressive behavior and a relatively favorable prognosis (44). The biological aggressiveness of classic PTC is partly influenced by underlying genetic alterations. For example, the *BRAF V600E* mutation is associated with more aggressive clinicopathological features, whereas receptor tyrosine kinase rearrangements are generally linked to comparatively less aggressive behavior (45). However, considering the limited number of cases, the prognostic impact ETC variants remains an important area for future investigation.

The management of cervical lymph nodes remains controversial (46). Our findings support therapeutic neck dissection when nodal metastasis is clinically or radiologically evident. However, the role of prophylactic lymph node dissection remains uncertain, particularly given the limited and heterogeneous evidence available from case reports and small case series. Similarly, the most controversial surgical decision concerns management of the orthotopic thyroid gland (40, 43, 46–48). Previous reports have generally supported a conservative approach in selected patients without orthotopic thyroid disease (49, 50). In our propensity-score-matched analysis, orthotopic thyroidectomy was not associated with reduced recurrence. Nevertheless, because the number of recurrence events in the matched cohort was small, this result should not be interpreted as definitive evidence of no benefit or clinical equivalence. Rather, the available case-based evidence does not support routine orthotopic thyroidectomy solely for presumed prognostic improvement. Surgical decision-making should therefore be individualized according to orthotopic thyroid findings, tumor location, histology, metastatic status, feasibility of surveillance, and patient-specific risk factors.

This study has several limitations. First, the relationship between iodine status and thyroid cancer development remains controversial. Some evidence suggests that excessive iodine intake may be an extrinsic risk factor for PTC (51). Specifically, excessive iodine intake, commonly defined as a urinary iodine concentration ≥300 μg/L, has been associated with an increased risk of PTC, whereas adequate iodine intake, defined as a urinary iodine concentration of 100–200 μg/L, may have a protective effect (52). In contrast, other studies have reported no significant association between questionnaire-estimated dietary iodine intake and thyroid cancer risk (53). In the studies included in our review, iodine status, including dietary intake, iodine deficiency, or supplementation, was generally not reported. Therefore, its potential role in the etiology of ETC could not be assessed.

Second, the search was restricted to English-language publications, which may have introduced language bias. Because ETC appears to be more frequently reported in Asian populations, this restriction may have led to underrepresentation of cases from non-English-speaking regions, particularly Asia. Third, the median follow-up duration of 27 months is relatively short for well-differentiated thyroid carcinoma, which may recur many years after initial treatment. Therefore, the recurrence rate reported in this review is likely underestimated. Fourth, missing data were common across the included reports, limiting the ability to perform complete multivariable analyses for several clinically relevant factors.

Several limitations in prognostic analysis should also be acknowledged. **i)** The relatively wide confidence interval for non-head and neck ETC (95% CI: 2.61–20.04) reflects the modest sample size of the thoracic, abdominal, and pelvic subgroup (n = 14). Nevertheless, the lower bound of the confidence interval remained well above 1.0, suggesting a consistently increased risk of death despite the limited sample size. **ii)** The age cutoff of 50 years was determined empirically, and its generalizability requires further validation. **iii)** Several subgroup analyses, including those stratified by tumor size, orthotopic thyroid status, and pathological type, were constrained by small sample sizes, low event counts, and imbalanced group distributions. **iv)** Considering merely a limited number of patients received radiotherapy, the association between radiotherapy and prognosis could not be reliably assessed. These limitations resulted in wide confidence intervals that crossed 1.0, reducing the precision of the corresponding effect estimates. Therefore, these findings should be interpreted cautiously and validated in larger cohorts.

Given the rarity of ETC, the available literature consists predominantly of case reports and small case series. Although this systematic review was conducted using a rigorous methodology, the evidence base does not fully satisfy all AMSTAR-2 or PRISMA 2020 standards, which are primarily designed for higher-level interventional or observational evidence (12, 15, 16). To strengthen methodological transparency, included case reports were appraised using the JBI critical appraisal checklist, and case series were evaluated using an established methodological quality framework (13, 14). Nevertheless, publication bias remains unavoidable. Case reports are more likely to describe unusual presentations, successful interventions, or clinically notable outcomes, whereas negative, inconclusive, or routine cases are less likely to be published. Consequently, the findings of this study should be interpreted as hypothesis-generating and should be validated in larger, multi-institutional registries or collaborative studies.

## Data Availability

The original contributions presented in the study are included in the article/**Supplementary Material**. Further inquiries can be directed to the corresponding authors.
