# Supplementary material for: The diagnosis and management of ectopic thyroid cancer: a systematic review
Source: Front Endocrinol (Lausanne). 2026 May 29;17:1856987. doi: 10.3389/fendo.2026.1856987 (PMC13259913; doi:10.3389/fendo.2026.1856987)
Supplement: Supplementary file 1 [file DataSheet1.docx]

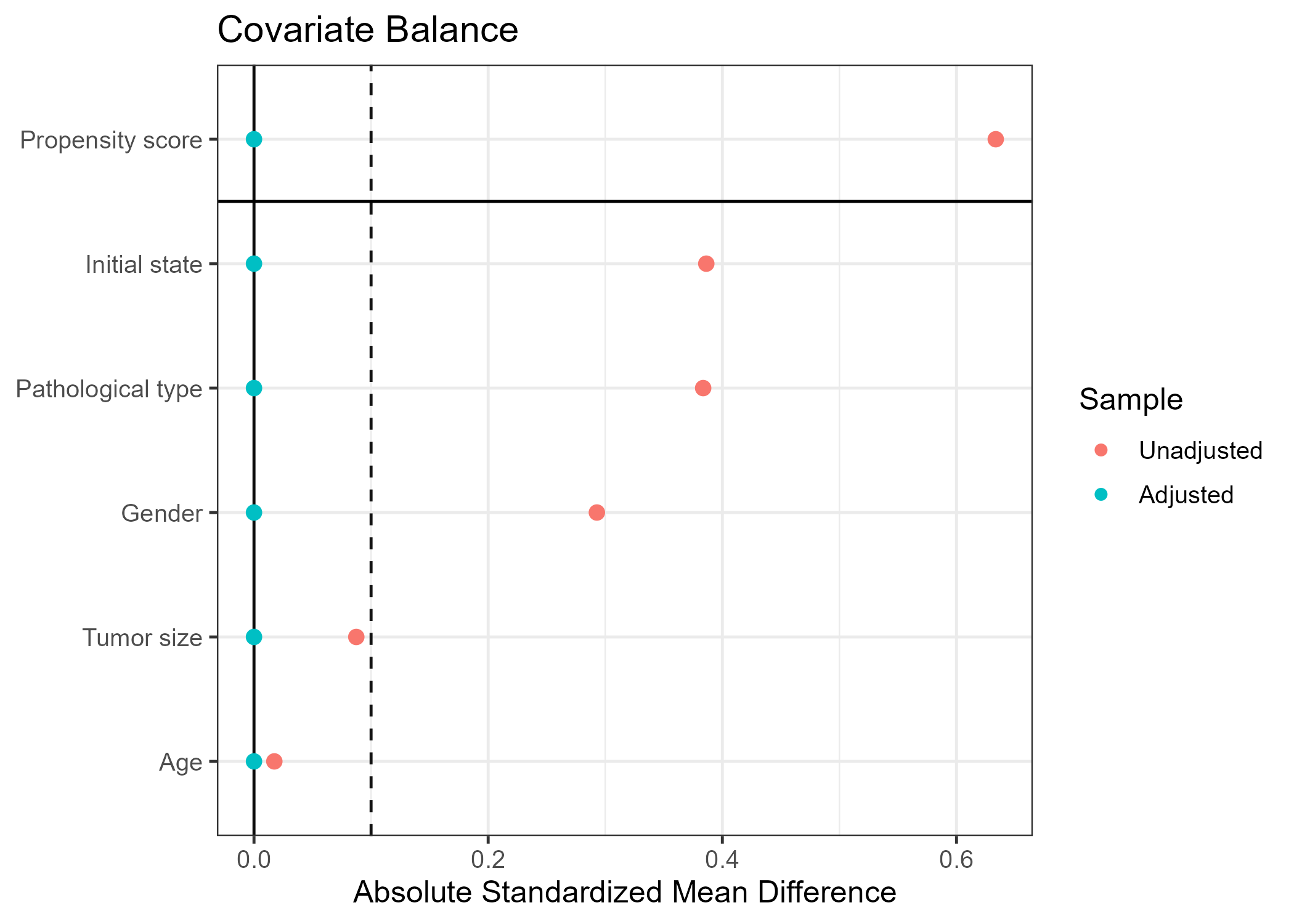


Supplementary Figure 1. Love plot of standardized mean differences. The figure shows the covariate balance before and after propensity score matching. Standardized mean differences are shown for covariates included in the propensity-score model. Exact matching was imposed on age and gender. All covariates achieved adequate balance, with standardized mean differences below 0.1.

Supplementary Table 1. Covariate balance before and after propensity score matching.

| Covariate | Comparison shown | SMD (pre-matching) | SMD (post-matching) |
| --- | --- | --- | --- |
| Age | >50 years vs. ≤50 years | 0.017 | <0.001 |
| Gender | Male vs. female | 0.293 | <0.001 |
| Initial state | Metastatic vs. localized | 0.386 | <0.001 |
| Pathological type | Other vs. PTC | 0.384 | <0.001 |
| Tumor size | >2 cm vs. ≤2 cm | 0.087 | <0.001 |

SMD, standardized mean difference.

Supplementary Table 2. Recurrence outcomes before and after propensity score matching.

| Analysis | Orthotopic thyroidectomy | No orthotopic thyroidectomy | P value | Statistical test |
| --- | --- | --- | --- | --- |
| Unadjusted full cohort（n=293） | 11.1% (22/199) | 10.6% (10/94） | 0.915 | Chi-square test |
| Propensity-score-matched cohort (n=50 per group) | 6.0% (3/50) | 4.0% (2/50) | 1.000 | McNemar test |

Supplementary Table 3. Clinical characteristics and treatment of recurrent patients

| Site | n | Age/Sex | Pathological  type | | Genetic Mutation | Disease stage | Orthotopic thyroid | Treatment | Neck dissection results | Recurrence time(months) | Recurrence site | Death after Recurrence |
| --- | --- | --- | --- | --- | --- | --- | --- | --- | --- | --- | --- | --- |
| Thyroglossal duct | 25 | 43^#^/16F,9M | | 21 PTC  4 ATC | 1 BRAF V600E | 14 Local  11 Metastatic | 8 Malignant  10 Benign  7 NA | 25 Surgery  15 Thyroidectomy  14 RAI  2 RAD  10 CLND | 8 Positive  2 Negative | 46^#^ | 14 LN  4 Local recurrence  3 Distant metastasis  4 NA | 7 Yes  18 No |
| Tongue | 4 | 52^#^/3F,1M | | 2 PTC  1 FTC  1 OCA | - | 4 Local | 1 Benign  3 Absence | 3 Surgery,  1 Thyroidectomy  2 RAI | - | 32^#^ | 1 LN  2 Local recurrence  1 Distant metastasis | 4 No |
| Lateral neck | 2 | 52^#^/2M | | 2 PTC | 1 BRAF V600E | 1 Local  1Metastatic | 2 Benign | 2 Surgery  2 Thyroidectomy  2 CLND | 1 Positive  1 Negative | 4^#^ | 2 LN | 2 No |
| Mid-neck | 2 | 69/2F | | 2 PTC | 1 BRAF V600E and TERT promoter(C228T) | 2Local | 2 Malignant | 2 Surgery  2 Thyroidectomy  1 RAI | - | 48 | 1 LN  1 Local recurrence | 2 No |
| Skull | 1 | 74/F | | FTC | - | Local | Benign | Surgery  Thyroidectomy | - | 55 | Local recurrence | Yes |
| Oropharynx | 1 | 53/F | | PTC | - | Local | malignant | Surgery | - | 9 | LN | No |
| Mediastinum | 2 | 66/2F | | 1 PTC  1 PDTC | - | 2 Metastatic | 2 Benign | 2 Surgery  2 Thyroidectomy  1 RAI  1 RAD  1 CLND | 1Positive | 5 | 2 Distant metastasis | 2 No |
| Thoracic spinal epidural space | 1 | 69/F | | PTC | - | Local | Benign | Surgery | - | 12 | Local recurrence | NO |
| Tracheal | 1 | 64/M | | PTC | - | Metastatic | Benign | RAD | - | 12 | LN | Yes |
| Substernal region | 1 | 67/F | | PTC | - | Local | Benign | Surgery  Thyroidectomy  CLND | Negative | 6 | Local recurrence | No |

n, number of studies; #, average; M, male; F, female; PTC, papillary thyroid carcinoma; ATC, anaplastic thyroid carcinoma; FTC, follicular thyroid carcinoma; OCA, oncocytic carcinoma; PDTC, poorly differentiated thyroid carcinoma; NA, not applicable; CLND: cervical lymph node dissection; RAI, radioactive iodine ablation; RAD, Radiotherapy; LN, Lymph node.

Supplementary Table 4. Clinical characteristics and treatment of deceased patients

| Site | n | Age/Sex | Pathological  Type | Genetic Mutation | Disease stage | Orthotopic thyroid | Treatment | Clinical course | Time of death | Cause of death |
| --- | --- | --- | --- | --- | --- | --- | --- | --- | --- | --- |
| The mandible | 1 | 80/F | FTC | - | Local | Benign | Surgery  Thyroidectomy | Died | 4 days after surgery | Unknow |
| Mediastinum | 1 | 95/F | ATC | - | Local | Benign | No | Died | 11 days after diagnosis | Respiratory failure |
| Mediastinum | 1 | 90/M | ATC | BRAF V600E | Metastatic | Absence | Excisional biopsy | Died | 6 months after primary diagnosis | Unknown |
| Porta hepatis | 1 | 79/M | FTC | - | Metastatic | Benign | Lenvatinib  Thyroidectomy | Died | 3 months after primary diagnosis | Metastatic disease |
| Thyroglossal duct | 1 | 66/M | PTC | - | Local | NA | Surgery | Died | NA | Liver metastases from the laryngeal cancer |
| Thyroglossal duct | 1 | 48/M | PTC | - | Local | Malignant | Surgery  Thyroidectomy  RAI | Died | After 8 years of follow-up | Myocardial infarction |

n, number of studies; M, male; F, female; PTC, papillary thyroid carcinoma; FTC, follicular thyroid carcinoma; ATC, anaplastic thyroid carcinoma; NA, not applicable.
